# The Psychoactive Effects of Psychiatric Medication: The Elephant in the Room

**DOI:** 10.1080/02791072.2013.845328

**Published:** 2013-11-18

**Authors:** Joanna Moncrieff, David Cohen, Sally Porter

**Affiliations:** a Mental Health Science, University College London, London, UK.; b University of California, Los Angeles, Los Angeles, CA.; c Addiction Psychiatry South London and Maudsley NHS Foundation Trust, London, UK.

**Keywords:** antidepressants, antipsychotics, prescription drug dependence, psychiatric drugs, psychoactive effects, withdrawal effects

## Abstract

The psychoactive effects of psychiatric medications have been obscured by the presumption that these medications have disease-specific actions. Exploiting the parallels with the psychoactive effects and uses of recreational substances helps to highlight the psychoactive properties of psychiatric medications and their impact on people with psychiatric problems. We discuss how psychoactive effects produced by different drugs prescribed in psychiatric practice might modify various disturbing and distressing symptoms, and we also consider the costs of these psychoactive effects on the mental well-being of the user. We examine the issue of dependence, and the need for support for people wishing to withdraw from psychiatric medication. We consider how the reality of psychoactive effects undermines the idea that psychiatric drugs work by targeting underlying disease processes, since psychoactive effects can themselves directly modify mental and behavioral symptoms and thus affect the results of placebo-controlled trials. These effects and their impact also raise questions about the validity and importance of modern diagnosis systems. Extensive research is needed to clarify the range of acute and longer-term mental, behavioral, and physical effects induced by psychiatric drugs, both during and after consumption and withdrawal, to enable users and prescribers to exploit their psychoactive effects judiciously in a safe and more informed manner.

A characteristic and well-recognized property of chemical substances used for recreational purposes is their ability to produce altered states of consciousness and concomitant changes in behavior by virtue of their action on the central nervous system. Drugs prescribed to treat psychiatric disorders, including drugs commonly classified as antidepressants, antipsychotics, anxiolytics, stimulants, and drugs such as lithium and anticonvulsants used to treat bipolar disorder, also modify normal mental processes and behavior, but there has been a widespread tendency to conflate these actions with a presumed effect on underlying disease processes. In this paper, we use the term “psychoactive effects” to refer to the way some substances produce altered cognitive and emotional states, which differ from the normal un-drugged state, and we distinguish these effects from the putative disease-specific effects of prescribed drugs. The distinction matters because, although significant, the consequences of the psychoactive effects of psychiatric medications are not well-recognized.

Just like the various substances that are used recreationally, each type of psychiatric medication induces a distinctive altered mental and physical state, whose characteristics depend largely on the nature of the drug ingested. Numerous studies with human volunteers and countless studies with animals document the range of ways that different psychiatric drugs impact on normal cognition, emotion, and behavior (Baldessarini 1985). Although the term “psychoactive” refers particularly to the mental alterations produced by drugs, most of these alterations appear intimately connected to physical or bodily effects, with many “mental” effects having concomitant physical manifestations, together producing a “global” drug effect. Sedation, for example, is both a mental and physical experience, and arousal, like that produced by stimulant drugs, has mental and physical aspects. It is likely that no psychoactive drug produces only mental effects.

Some psychiatric medications produce pleasurable psychoactive effects, or euphoria, and have consequently become drugs that some people use recreationally and sometimes excessively (leading to their designation as possessing “abuse potential”). This has been the fate of stimulants like amphetamine, introduced as a treatment for depressive neurosis in the 1940s (Rasmussen 2006). Apart from their use in attention-deficit hyperactivity disorder, they are now most commonly associated with recreation and performance enhancement. Benzodiazepines and the related “Z-drugs” continue to be widely prescribed in general practice and psychiatry, but have become popular black market drugs, frequently used alongside opiates by those with serious addiction problems. Anecdotal evidence suggests that some other psychiatric medications, including quetiapine and amitriptyline, have a modest “street” value for their sedative effects.

Other psychiatric drugs are more often associated with the experience of sharply unpleasant psychoactive effects or dysphoria. This is most notably the case with the neuroleptic or antipsychotic drugs, but selective serotonin reuptake inhibitors (SSRIs), tricyclics, and lithium are also generally disliked by volunteers (Dumont et al. 2005; Judd et al. 1977). The fact that these drugs are not associated with euphoria, and therefore do not usually induce craving or become drugs of abuse, does not make them any less “psychoactive” than recreational drugs, nor does it exclude them from inducing physical dependence.

When novel drugs were introduced into psychiatry in the 1950s, in contrast to nowadays, clinicians and researchers expressed considerable interest in their characteristic mind- and behavior-altering effects. They described the striking state of mental restriction provoked by the early neuroleptics, for example, which they contrasted with the more familiar type of sedation produced by barbiturates:

…the apparent indifference, or delay in response to external stimuli, the emotional and affective neutrality, the decrease in both initiative and preoccupation without alteration of conscious awareness or in intellectual faculties, constitute the psychic syndrome due to treatment (Delay & Deniker 1952, 503–504).

Reports of early antidepressants, such as iproniazid, also described their immediate psychoactive effects, which appeared similar to those of stimulants (Crane 1956). Few published accounts have described the subjective effects induced by the many other drugs used as antidepressants, however. Existing evidence suggests that tricyclics are strongly sedative and also dysphoric (Dumont et al. 2005; Herrmann & McDonald 1978). SSRIs and venlafaxine appear to produce a state of lethargy and indifference, coupled with an unpleasant state of agitation, tension, and hostility in some people (Goldsmith & Moncrieff 2011). An in-depth qualitative study describes the “essential lived characteristic” of being on SSRIs as “increased distance or disconnection between takers [and] their worlds” (Teal 2009, 19). Lithium produces dysphoria, lethargy, and cognitive slowing and impairment in volunteers (Judd et al. 1977; Squire et al. 1980). Anticonvulsants, today part of the standard maintenance treatment of bipolar disorders, show a panoply of psychoactive effects, ranging from strong sedation and cognitive slowing to anxiety and agitation (Cavanna et al. 2010).

Although the immediate effects of common psychoactive drugs are best known (e.g., the intoxication produced by alcohol), the continued use of drugs with central nervous system activity has further consequences for mental functioning. Firstly, tolerance to some immediate effects may develop, and secondly, additional mental or emotional alterations may occur, either as a direct result of the continued presence of the drug in the body, or of the body's delayed adaptations to it. Tolerance is known to develop to at least some effects of most recreational substances. Though it has been little investigated in relation to classes of drugs used in psychiatry, animal research demonstrates that bodily adaptations develop within days of continuous use of the antipsychotic drug haloperidol, for example (Samaha et al. 2007). Late-onset mental state changes are more difficult to ascribe with certainty to drug ingestion, but we know that chronic stimulant use can produce psychotic states, and long-term alcohol use is associated with depression (Schuckit 1994), and some evidence suggests long-term use of benzodiazepines is associated with dementia (Billioti et al. 2012). The possibility of adverse psychological and behavioral effects occurring after long-term use of other psychiatric medications has periodically been suggested (Barnhart et al. 2004; Myslobodsky 1993) but has received little attention, despite the reasonable argument that persistent use of any drug that produces alterations of normal mental and physical states should be expected to have long-term consequences.

Furthermore, discontinuation of most psychoactive substances after chronic use produces mental and physical changes. Withdrawal from psychiatric medications, including antidepressants and antipsychotics, is associated with distinctive withdrawal or discontinuation syndromes, which are suppressed by resumption of the drug (Judah et al. 1961; Lejoyeux & Ades 1997). Within different drug classes, drugs with a short half-life (including paroxetine, venlafaxine, and clozapine) typically provoke the most intense withdrawal symptoms (Goudie et al. 1999; Hindmarch et al. 2000). Users own reactions’ to the pharmacological effects of long-term drug use and subsequent withdrawal add another layer of consequences. As with several other psychoactive drugs, withdrawal symptoms might be the most distinctive component of some people's psychiatric medication experience.

## PSYCHOACTIVE EFFECTS AND MENTAL SYMPTOMS

People use licit substances like caffeine, alcohol, and nicotine to achieve a range of effects, including enhancing performance and sociability, producing relaxation, and managing stress or everyday emotional discomfort. People also sometimes use licit and illicit substances to combat more severe symptoms of anxiety and depression, to suppress painful memories of trauma, and to help manage or “escape” from psychologically or physically challenging situations, like living on the street or dealing with chronic stressors. Anecdotally, people are reported to have used illicit substances like opiates to self-medicate psychotic symptoms. Whether using drugs is ultimately helpful or harmful to the user depends on a variety of interacting factors, including the reason for using, the quantity taken, the manner and duration of use, the characteristic physical and mental effects the drug produces, the situation and personality of the individual user, the habits and lifestyle associated with the drug's use, as well as the social attitudes and legal penalties attached to it.

The psychoactive properties of prescribed psychiatric drugs also interact with the symptoms of mental disorders, again in ways that depend at least on the nature of the symptoms, the properties of the drug, and the social circumstances of the user. For example, the particular quality of physical, mental, and emotional restriction produced by neuroleptic or antipsychotic drugs was well recognized in the 1950s to dampen intense psychotic thoughts and experiences and other states of agitation and arousal, without simply putting the patient to sleep (Deniker 1960; Flugel 1959). This theory of neuroleptic action is supported by more recent research that confirms that antipsychotic drug treatment more commonly reduces the intensity or “salience” of psychotic symptoms, rather than removing them altogether (Mizrahi et al. 2005). Such results are confirmed by patients’ descriptions of how the psychoactive effects of antipsychotics reduce psychotic symptoms while also suppressing other aspects of mental activity (Moncrieff et al. 2009). These characteristic effects of neuroleptics make them usually effective, although not necessarily safe, agents for the practice of rapid tranquilisation (TREC Collaborative Group 2003) and for the control of symptoms of mania (Prien, Caffey & Klett 1972). These effects may also be seen as useful in other circumstances involving impulsive and aggressive behavior where alternative strategies are lacking or difficult to implement, including use in people with dementia or people who are considered personality disordered. Tolerance and other factors may counteract their effects in some of these situations, however (Maher et al. 2011). The ability to flatten emotions may explain why neuroleptics are distinguished from placebo in trials of depression (Robertson & Trimble 1982), and this, combined with their sedative effects, would also explain why many people find them helpful in anxiety (Maher et al. 2011). The usefulness of neuroleptics is limited by their distinctly dysphoric effects, however, as well as their serious physical complications, which would be expected to tilt the balance against their use for all but the most severely disabling conditions. Other drugs with sedative properties may be equally useful in some of these situations. Thus, acute mania is known to respond to lithium and sodium valproate (Bowden et al. 1994) and to benzodiazepines (Chouinard 1988).

Drugs commonly called antidepressants produce various psychoactive effects. The sedative effects of tricyclics may be useful for insomnia, anxiety, or agitation and these effects are not restricted to people with a diagnosis of depression, as reflected in the continuing popularity of low-dose tricyclic prescribing (Ilyas & Moncrieff 2012). Benzodiazepines may be preferable on safety grounds, however, if temporary sedation is what is intended. The benefits of other antidepressants are not so clear. The emotional flattening or disengagement described in relation to SSRIs may reduce feelings of depression, but the generalized nature of this effect, and its association with loss of libido (Goldsmith & Moncrieff 2011), would argue against the utility of these drugs in depression. We can speculate on how other psychoactive drugs — such as amphetamines, which induce euphoria and were long used as antidepressants (Rasmussen 2006), and opiates, which produce emotional anesthesia (Savvas et al. 2012) — might reduce or mask depressive symptoms. Drugs that produce short-term mood elevation, however, typically require increasing dose to maintain this effect and entail dysphoria when they are discontinued. No known substance appears capable of producing long-term mood elevation, which hints at the misleading nature of the term “antidepressant” (Moncrieff & Cohen 2006).

Use of psychiatric drugs is only worthwhile if the benefits outweigh the harms. Calculating a harm/benefit ratio is a complex undertaking, however, given that what is considered harmful or beneficial varies according to many factors, including the perspective of the observer and the phase of treatment. Individuals will also have different subjective responses to prescribed drugs, just as people respond differently to recreational substances. The lack of data about the consequences of the long-term use of prescribed psychiatric medications on the full range of human emotions and cognitive functions further hampers a thorough and balanced assessment of their value or otherwise, especially since they are normally prescribed for months and often for years. Moreover, it is often the subtle and easily overlooked aspects of drug treatment that users find most troubling. The mental effects of antipsychotics, for example, can be experienced as more unpleasant and impairing than their physical effects, and can interfere with people's ability to carry out daily tasks (Awad 1993; Moncrieff et al. 2009). In a study of SSRIs, mental effects led to drug discontinuation as often as physical effects (Bolling & Kohlenberg 2004).

On the other hand, some people actively seek the mind-altering properties of prescribed or illicit substances in order to manage distressing emotional states, and they may use both sorts of drugs simultaneously. Use of multiple prescribed and illicit psychoactive substances risks producing compound and unpredictable effects on normal functioning and symptoms. Although there is a general recognition that the use of recreational substances can become detrimental to a person's mental as well as physical health, such effects are rarely considered when psychiatric medications are prescribed. It is clear that the mind-numbing effects of psychoactive drugs like opiates and alcohol can constitute a barrier to addressing underlying psychological or personal issues, or environmental adversity, and to forming the supportive social relationships that improve the chances of long-term recovery. Just as substance misuse services encourage people to identify what function their drug use satisfies, and to develop less harmful strategies for dealing with problems, it may be preferable for psychiatric services, in some instances, to help individuals identify alternative ways of coping with emotional distress rather than prescribing medication. As with the misuse of recreational substances, identifying the function the drug fulfills and the pharmacological effects that facilitate its function may help people to develop alternative strategies for coping with adverse emotions and experiences.

## PSYCHOACTIVE EFFECTS AND MECHANISM OF ACTION

Drugs with psychoactive effects, whether taken for therapeutic or recreational purposes, will affect the thoughts, feelings, and behaviors that are constitutive of conditions referred to as mental disorders. They will therefore impact on self- or clinician-reported measures of psychological symptoms in placebo-controlled trials. Yet because these psychoactive effects have not been controlled or accounted for in the vast majority of clinical studies of psychiatric medications, no one is in a position to state whether these drugs have, in fact, any other type of reliable action relevant to the treatment of symptoms. In particular, we do not know whether psychiatric medications also modify the still-unknown physiological or biochemical processes that are hypothesized to give rise to the symptoms of particular mental disorders as delineated in current diagnostic manuals. We do not have evidence that psychiatric drugs exert targeted, disease-specific effects.

Studies that compare a drug thought to have a disease-specific action with a drug that produces similar psychoactive effects but is not thought to act on the disease process might be able to clarify how a drug “works.” Even then, there would remain the question of whether the altered states produced by the two drugs were really equivalent. In any case, there have been few such studies and those that exist provide little confirmation that psychiatric drugs exert a disease-specific action, independent of their psychoactive properties. Two early studies found that antipsychotics were superior to a barbiturate in people with psychosis, but it had already been noted that antipsychotics produce a different sort of altered state from barbiturates (Casey et al. 1960a; Casey et al. 1960b). Two studies compared lithium to antipsychotics for the treatment of various psychotic states and neither showed any difference between the drugs’ effects on people with different diagnoses (affective psychosis compared with schizophrenic disorders) (Braden et al. 1982; Johnstone et al. 1988). Although one of the studies claimed to show a differential effect on individual symptoms, this was only achieved through a complex analysis that left numbers small and most differences statistically non-significant, and did not compare effects of the drugs directly (Johnstone et al. 1988). Overall, several old studies comparing opiates and benzodiazepines with antipsychotics for psychosis were unable to distinguish the different sorts of drugs (Abse et al. 1960; Wolkowitz & Pickar 1991). Clinical trials with a wide array of psychoactive substances that are not usually thought of as antidepressants, including stimulants, benzodiazepines, and neuroleptics, show effects on depression rating scales similar to those produced by drugs classified as antidepressants (Moncrieff 2001). On the other hand, the idea that a delay occurs between the start of psychiatric medication and the subject's therapeutic response might argue against immediate psychoactive effects of psychiatric medication explaining part of the therapeutic effects rated in clinical trials. However, recent analyses of antipsychotic and antidepressant trials suggest that drug effects occur quite early (Agid et al. 2003; Walsh et al. 2002).

“Amplified” placebo effects may also contribute to drug-placebo differences in randomized controlled trials (Moncrieff, Wessley & Hardy 1998). Apart from their direct action on symptoms, psychoactive and physical effects may reveal to researchers and participants in placebo-controlled trials who is receiving active medication and who is not, causing the placebo effect of medication to be amplified and to exceed that produced by inert placebo tablets. Placebo-controlled studies cannot therefore in principle establish whether a drug works, if it does, by reversing an underlying pathological process or by inducing an altered mental and physical state which may directly affect manifestations of the disorder or subvert the double-blind design of clinical trials.

## DEPENDENCE

It is now accepted that all major classes of psychiatric medication produce distinctive withdrawal effects which mostly reflect their pharmacological activity. These effects are significant not only because they can prevent someone from stopping medication when they do not need it or want it anymore, but also because they may be—and probably often are—mistaken for signs of relapse (Moncrieff 2006). This creates a situation whereby patients become psychologically as well as physically dependent on their medication, since they (and their prescribers too) may come to believe that they cannot manage without it.

That drugs like antidepressants and antipsychotics are being prescribed for longer and longer periods suggests that some people may find it difficult, either for physical or psychological reasons, to stop medication once it is started (Moore et al. 2009; Prah et al. 2012). This is of particular concern given the serious adverse consequences associated with long-term use of drugs like antipsychotics, which include cardiac complications, metabolic dysfunction, and neurological damage such as tardive dyskinesia (Newcomer & Haupt 2006; Ray et al. 2009; Tarsy et al. 2011). More help is needed to support people who wish to stop psychiatric medication when it is considered safe to do so, and further research should clarify the full range of withdrawal effects and their likely duration, since there are reports of protracted and disabling withdrawal states following the discontinuation of some prescribed drugs (Modell 1997; Precourt et al. 2005). General physicians and healthcare workers need more information and training about devising tapering schedules, recognizing withdrawal-related symptoms and distinguishing them from prior symptoms, in order to improve their confidence and ability to support people who wish to withdraw from prescribed medication (Cohen 2007). Therapy focusing on finding alternative techniques for managing emotional states such as that provided in drug and alcohol rehabilitation programs may be necessary for people who have been on mind-, mood-, and behavior-altering drug treatment for long periods.

## CONCLUSIONS

Approaching psychiatric medications as drugs which produce immediate and delayed psychoactive effects, and which induce tolerance and dependence, fundamentally differs from the conventional understanding that suggests these drugs exert specific actions on (presumed) underlying disease processes. According to the conventional view, the drugs’ psychoactive properties are merely incidental “side-effects.” Despite six decades of intensive research in neuropharmacology, however, there is a lack of evidence that psychiatric drugs have a disease-specific action independent of their demonstrable psychoactive effects. These facts suggest that a radical change of thinking may be necessary about the nature, possibilities, and limitations of psychiatric drug treatment. Lessons from the use and misuse of other psychoactive substances can help to enlighten us about the broad range of behavioral effects that different psychiatric medications are likely to exert, and how these effects might interact with the psychological, behavioral, and other problems we call mental disorders.

This reorientation would demand that people prescribed psychiatric medicaments are treated as informed consumers, rather than passive recipients of diagnosis-driven prescribing. The user's subjective experience should guide the use of psychiatric medications in a collaborative dialogue with the prescriber, rather than changes in designated symptoms or clusters of symptoms. Much more information is required, however, on the behavioral pharmacology of psychiatric drugs, including all the mental, behavioral, and physical effects they induce in the acute phase and long-term, and during consumption and withdrawal. Data of this sort is likely to lead to revision of manufacturer-recommended dosing schedules which take no account of the quantitative relationship between dose and subjectively-experienced effects, derived as they are from fixed-dose studies and from trials narrowly focusing on clinician-rated target symptoms. We also need to assess further how the various drug-induced effects might interact with particular psychological symptoms in different circumstances and from the point of view of different observers. Only when we appreciate the nature of psychiatric drugs as psychoactive substances can we start to accumulate the knowledge necessary to enable prescribers and consumers to use these drugs safely and effectively.

Re-orienting drug therapy in this manner also raises questions about the validity and relevance of diagnostic systems such as the recently published DSM-5. The idea that psychiatric pharmaceuticals exert a disease- or disorder-specific action has long been one of the principal justifications for modern classification (Spitzer 1976), but as we indicate above, there is in fact no compelling evidence to support this supposition. Using psychiatric drugs explicitly for their psychoactive effects implies the need for a different understanding of the nature of psychiatric problems, one that focuses not on diagnoses or syndromes that are presumed to represent the manifestations of a discrete underlying pathology, but to an individualized appreciation of the nature, context, and origins of each person's particular behavioral and emotional difficulties. Such an approach would break the alleged link between diagnoses and treatment, and enable a frank discussion about the purpose and ethics of the already frequent “off-label” use of prescribed psychoactive medications, such as their use for behavioral control in children and the elderly.

Acknowledging the psychoactive effects of psychiatric medication naturally also raises the thorny question of why some psychoactive drugs are prohibited and others not. Although this topic is beyond the scope of this paper, we note that some drugs which are used recreationally for their euphoric effects appear to be useful agents in various situations: benzodiazepines for their effects on emotional and behavioral disturbance; stimulants for the performance of tasks requiring unusual endurance; and possibly hallucinogens, when people seek insights in therapeutic situations or during end-of-life care (Grob et al. 2011). When the nature of the useful effect is identified, however, other non drug-based ways of achieving the same result may be devised that avoid the potentially harmful consequences of drug exposure (Macready 2012). Similarly, recognizing the psychoactive effects of psychiatric medications may facilitate the development of alternative strategies for ameliorating mental distress and also draw attention to some potentially anti-therapeutic consequences of using psychoactive substances as therapeutic agents.
